# Clinical feasibility of an advanced neonatal epidermal multiparameter continuous monitoring technology in a large public maternity hospital in Nairobi, Kenya

**DOI:** 10.1038/s41598-022-16051-3

**Published:** 2022-07-09

**Authors:** Amy Sarah Ginsburg, Sahar Zandi Nia, Dorothy Chomba, Millicent Parsimei, Dustin Dunsmuir, Mary Waiyego, Jesse Coleman, Roseline Ochieng, Guohai Zhou, William M. Macharia, J. Mark Ansermino

**Affiliations:** 1 Clinical Trials Center, University of Washington, Seattle, Building 29, Suite 250, 6200 NE 74th Street, Seattle, WA 98115 USA; 2 Department of Anesthesiology, The University of British Columbia, Vancouver, BC Canada; 3 Department of Pediatrics, Aga Khan University, Nairobi, Kenya; 4 Pumwani Maternity Hospital, Nairobi, Kenya; 5 Evaluation of Technologies for Neonates in Africa, Seattle, USA; 6 Center for Clinical Investigation, Brigham and Women’s Hospital, Boston, MA USA

**Keywords:** Health care, Medical research

## Abstract

Clinically feasible multiparameter continuous physiological monitoring technologies are needed for use in resource-constrained African healthcare facilities to allow for early detection of critical events and timely intervention for major morbidities in high-risk neonates. We conducted a prospective clinical feasibility study of a novel multiparameter continuous physiological monitoring technology in neonates at Pumwani Maternity Hospital in Nairobi, Kenya. To assess feasibility, we compared the performance of Sibel’s Advanced Neonatal Epidermal (ANNE) technology to reference technologies, including Masimo’s Rad-97 pulse CO-oximeter with capnography technology for heart rate (HR), respiratory rate (RR), and oxygen saturation (SpO_2_) measurements and Spengler’s Tempo Easy non-contact infrared thermometer for temperature measurements. We evaluated key performance criteria such as up-time, clinical event detection performance, and the agreement of measurements compared to those from the reference technologies in an uncontrolled, real-world setting. Between September 15 and December 15, 2020, we collected and analyzed 503 h of ANNE data from 109 enrolled neonates. ANNE’s up-time was 42 (11%) h more for HR, 77 (25%) h more for RR, and 6 (2%) h less for SpO_2_ compared to the Rad-97. However, ANNE’s ratio of up-time to total attached time was less than Rad-97’s for HR (0.79 vs 0.86), RR (0.68 vs. 0.79), and SpO_2_ (0.69 vs 0.86). ANNE demonstrated adequate performance in identifying high and low HR and RR and high temperature events; however, showed relatively poor performance for low SpO_2_ events. The normalized spread of limits of agreement were 8.4% for HR and 52.2% for RR and the normalized root-mean-square deviation was 4.4% for SpO_2_. Temperature agreement showed a spread of limits of agreement of 2.8 °C. The a priori-identified optimal limits were met for HR and temperature but not for RR or SpO_2_. ANNE was clinically feasible for HR and temperature but not RR and SpO_2_ as demonstrated by the technology’s up-time, clinical event detection performance, and the agreement of measurements compared to those from the reference technologies.

## Introduction

In high-income countries, multiparameter continuous monitoring technologies can be essential in the clinical management of high-risk neonates. However, these technologies are frequently unavailable in resource-constrained African settings despite higher neonatal morbidity and mortality^1,2^. At a large, public, referral hospital in Nairobi, few neonates had vital signs recorded within the first hour of life, and less than half received heart rate (HR; 44%), respiratory rate (RR; 44%) or temperature (46%) recordings on their first day of hospital admission^3^. Nursing shortages and high patient workloads at six hospitals in Nairobi county have been associated with missed vital signs monitoring and other nursing tasks for sick neonates^4^. Neonatal multiparameter continuous monitoring technologies could facilitate and improve quality of care by expanding nurses’ capacities to reliably and efficiently monitor more neonates. Neonatal innovations appropriate for use in resource-constrained health facilities are needed to allow for early detection of critical events and timely intervention for major morbidities^5,6^.

Sibel’s Advanced Neonatal Epidermal (ANNE) wireless, multiparameter, continuous monitoring technology includes two neonatal-sized, non-invasive, adhesive skin sensors attached directly to the skin surface that are capable of continuously measuring and recording HR, RR, oxygen saturation (SpO_2_), and skin surface temperature (Supplementary Fig. S1). Up to 30 h of data can be stored within the sensor and transmitted wirelessly to a central database supported by customized software. The projected cost of commercial acquisition of Sibel’s reusable and rechargeable technology is about $40USD with an additional $0.20USD per unit for adhesives/consumables lasting 24 to 72 h. In a previous clinical trial of neonates in Kenya, we evaluated the accuracy of ANNE to measure HR, RR, SpO_2_, and temperature when compared to verified reference technologies^7^. We also completed qualitative assessments of the feasibility, usability, and acceptability among healthcare personnel and caregivers by conducting in-depth interviews and observations^8,9^.

Novel medical technologies may compare favorably to established reference technologies in more controlled research settings; however, also important to ascertain is their clinical feasibility in uncontrolled, real-world settings. If clinical feasibility performance is not evaluated and the findings incorporated during technology development and refinement, a novel medical technology’s adoption, uptake, scale-up, and use in clinical practice may be impacted. Thus, in this study we evaluated key performance criteria such as up-time (periods of adequate signal quality), clinical event detection performance, and the agreement of measurements compared to those from the reference technologies in an uncontrolled, real-world setting.

## Methods

### Study design and participants

We conducted a prospective, observational, clinical feasibility study of ANNE at Pumwani Maternity Hospital (PMH) in Nairobi, Kenya, the largest referral public maternity hospital in sub-Saharan Africa. PMH has no neonatal intensive care unit. Caregivers of neonates delivered at or admitted to PMH were approached by trained study staff who obtained written informed consent and assessed the neonate for study eligibility based on the results of the medical history, clinical examination, and appropriate understanding of the study by the caregiver (Table 1). Carried out in accordance with the Declaration of Helsinki and Guideline for Good Clinical Practice/ International Standards Organization (ISO) 14155 to ensure accurate, reliable, and consistent data collection, the study protocol was approved by Western Institutional Review Board (20191102), Aga Khan University Nairobi Research Ethics Committee (2019/REC-02), and Kenya Pharmacy and Poisons Board (ECCT/19/05/02) and registered with ClinicalTrials.gov, NCT03920761^10^. Effort was made to ensure study participation did not interfere with or unnecessarily delay clinical care of neonates.

**Table 1:** Study eligibility criteria, endpoints, and definitions.

**Eligibility criteria**	
Inclusion	Neonate with corrected age of < 28 days requiring admission to the neonatal ward, the post-natal ward, or the neonatal high dependency unit at Pumwani Maternity Hospital for prematurity or other clinical indication(s) based on the attending physician’s assessment Caregiver(s) willing and able to provide informed consent and available for follow-up for the duration of the study
Exclusion	Receiving continuous positive airway pressure or mechanical ventilation Skin abnormalities in the nasopharynx and/or oropharynx Contraindication to skin sensor application Known arrhythmia Congenital abnormality requiring major surgical intervention Any medical or psychosocial condition or circumstance that would interfere with study conduct or for which study participation could put the neonate’s health at risk
**Study endpoints**	
Up-time duration of ANNE compared to the reference technologies Diagnostic performance of ANNE compared to the reference technologies for clinical event detection including sensitivity, specificity, positive predictive value, negative predictive value, and ratio of false negative-to-false positive events Agreement between ANNE and the reference technologies for heart rate (HR), respiratory rate (RR), oxygen saturation (SpO_2_), and temperature	
**Study definitions**	
Total time attached	Measured in minutes as non-zero values recorded by the technology starting 10 min after technology placement and 5 min before disconnection; the 5-min periods before temporary removal and after reconnection to ANNE and the reference technologies were also excluded
Up-time	Measured in minutes as the total time the sensor was attached that met the a priori-identified *signal quality* limits for each technology
Signal quality	*HR and SpO*_*2*_ ANNE—for every second, we evaluated the preceding 59 s in addition to the current second to ensure that all 60 (100%) seconds > 0 Rad-97—for every second, we evaluated the preceding 59 s in addition to the current second to ensure that at least 30 (50%) seconds demonstrated a signal quality index (Masimo SQI) > 150 *RR* ANNE—for every second, we evaluated the preceding 59 s in addition to the current second to ensure that all 60 (100%) seconds > 0 Rad-97—for every second, we evaluated the preceding 59 s in addition to the current second to ensure that at least 30 (50%) seconds demonstrated no capnography exceptions, indicating low RR quality, and a capnography quality score ≥ 2 *Temperature* ANNE—all temperatures > 0 Spengler’s technology—all temperature spot checks
Event second	Any second that contains a high or low HR or RR event (a value above or below the thresholds) for either ANNE or Rad-97
Event window	A 10-min window centered from 5 min before to 5 min after the first *event second* noted by the reference technology; no overlapping windows are allowed, so *event seconds* less than 5 min from the end of the previous *event window* result in the new *event window* starting immediately following the previous window
True positive event	A reference technology *event window* containing at least 1 *event second* identified by ANNE
False negative event	A reference technology *event window* containing no *event seconds* recorded by ANNE
False positive event	An event recorded by ANNE outside all reference technology’s *event windows*
True negative event	Any 10-min window with no events recorded by either ANNE or the reference technology
Clinically significant event	Any *false negative* or *false positive event* that would likely require a clinician to institute a change in clinical practice

### Study procedures

Enrolled neonates received local standard of care while additionally being simultaneously monitored with the ANNE multiparameter, continuous monitoring technology (Sibel Inc. IL, USA), the Rad-97 pulse CO-oximeter with capnography (Masimo Corporation, USA), and Spengler’s Tempo Easy non-contact infrared thermometer (SPENGLER HOLTEX Group, Aix-en-Provence, France). The Rad-97 was selected as a reference technology based on its ability to extract and record high resolution data, perform neonatal capnography and pulse oximetry, and its compact design enabling bedside monitoring. Tempo Easy was chosen as a reference technology for temperature monitoring due to its in-country availability. HR, RR, and SpO_2_ data were collected in real-time for a minimum of one hour from ANNE and Rad-97 and temperature data via hourly spot checks with the Tempo Easy technology. The total opportunity for attaching the technologies was similar. Up-time, clinical event detection performance, and HR, RR, SpO_2_, and temperature measurements data were collected by ANNE and the reference technologies (Table 1). For temperature, readings from ANNE’s chest sensor were compared to Tempo Easy’s skin temperature readings from the forehead.

### Outcomes

To ensure objective measures of clinical feasibility, study outcomes included comparisons between the ANNE and reference technologies’ total time attached and up-time, and event detection of high and low HR and RR events, low SpO_2_ events, and high temperature events. In addition, we evaluated the agreement between HR, RR, SpO_2_, and temperature measurements in a real-world setting (Table 1).

### Data processing and analysis

The total number of minutes the sensors were attached and the up-time for each technology were calculated. We assessed the quality of the measurements from the ANNE and reference technologies, and designated periods of adequate signal quality data as up-time (Table 1). For ANNE, we obtained HR, RR, and SpO_2_ measurements with their corresponding proprietary signal quality index every second. Raw data collected in real-time was retrieved from the Rad-97 with a custom Android application. Data were parsed in C (Dennis Ritchie & Bell Labs, USA) to obtain plethysmograph waveform and plethysmograph quality index (PO-SQI) data at 62.5 Hz (Hz), and capnography (carbon dioxide (CO_2_)) waveform data at approximately 20 Hz. CO_2_ waveform data were analyzed using a breath detection algorithm developed in MATLAB (Math Works, USA) based on adaptive pulse segmentation^11^. We obtained HR and RR from intra-beat and breath duration intervals, respectively. A custom algorithm based on capnography features was utilized to determine the capnography quality index (CO_2_-SQI). SpO_2_ values were calculated by the Rad-97 at 1 Hz, and 8-s medians were used in the analysis. To standardize event detection, upper limits for HR and RR were individualized for each neonate and were calculated for HR to be 20% and for RR to be 15% greater than their respective baseline values (based on a review of historical data) once the neonate was settled (approximately 15 min after monitoring was started) but no less than 140 beats/minute for HR and 40 breaths/minute for RR. For all neonates, an upper limit temperature of 37.5 °C was used. Lower limits were static with values of 80 beats/minute for HR, 15 breaths/minute for RR, and 90% for SpO_2_ based on the normal physiological range. To identify clinical events, Sibel provided a software parser that processed all of their monitoring data and provided event detection in pseudo- real-time that was finalized prior to data collection. Reference technology data were processed following data collection with a custom algorithm to identify events.

For overlapping periods of up-time from ANNE and Rad-97, clinical events were detected by examining the previous minute of data for both technologies. An event was identified if the one-minute median and the most recent ten-second median both met adequate signal quality and exceeded the threshold for upper or lower alarm values for HR, RR, SpO_2_, or temperature. Using a custom algorithm, events were aggregated from the technologies into ten-minute windows categorized as true positive, false negative, false positive, or true negative for high and low HR and RR and low SpO_2_ for each variable (Table 1)^12^. A panel of trained neonatologists and senior neonatology fellows conducted a manual adjudication of algorithmically-identified false negative and false positive events to identify those that were clinically significant (Table 1). For HR and RR, all false negative and false positive events that were greater than ten beats per minute for greater than two minutes were submitted for adjudication. For SpO_2_, all false negative and false positive events that had a difference greater than 4% between ANNE and Rad-97 for greater than two minutes were submitted for adjudication. Adjudication was not conducted for temperature monitoring due to a low number of events. Adjudication packages consisting of visualizations of selected false negative and false positive HR, RR, and SpO_2_ events and adjudication checklists were developed and provided to the panel of adjudicators (Supplementary Fig. S2). All false negative and false positive events were independently evaluated by two adjudicators and a third adjudicator reconciled the results if there was disagreement. To evaluate ANNE’s event detection, confusion matrices were generated and performance parameters including sensitivity, specificity, positive predictive value (PPV), negative predictive value (NPV), and false negative-to-false positive ratio for high and low HR and RR, low SpO_2_, and high temperature were calculated for both pre- and post-adjudication results.

We evaluated measurement agreement between ANNE and the reference technologies as the normalized bias and the normalized spread between the 95% limits of agreement (LOA) calculated by dividing the bias and spread between the 95% LOA by the overall reference HR, RR, SpO_2_, or temperature mean value^13^. We also calculated the root-mean-square deviation (RMSD) for each comparison. Based on the reference technology verification phase, acceptable a priori-identified normalized spreads between the 95% upper and lower LOA of 30% were selected for both RR and HR^14^. For SpO_2_, the adequate a priori-identified limit for RMSD was 4.5% and the optimal limit was 3.5% based on standards from the International Organization for Standardization^15^. For temperature, an adequate a priori-identified limit of 4.5 °C for the LOA was selected based on a literature review and a formal validation study^7^. R version 4.0.2 (Vienna, Austria 2020) was used for the data analysis.

### Role of the funding source

The funders of the study had no role in the study design, data collection, data analysis, data interpretation, or writing of the report.

## Results

Between September 15 and December 15, 2020, we enrolled 114 neonates, 109 of whom were included for analysis (Supplementary Fig. S3). Five neonates were excluded due to less than one hour recording duration with ANNE. Neonates were enrolled from the neonatal ward (101; 92.7%), the postnatal ward (5; 4.6%), and the neonatal high dependency unit (3; 2.8%). The median estimated age of the 57 males and 52 females was less than 24 (interquartile range less than 24, 120) h and the gestational age was 39 (range 37, 40) weeks. The median weight was 3.0 (range 2.5, 3.3) kilograms. Common primary and secondary diagnoses included sepsis or suspected sepsis (29; 26.6%), respiratory distress syndrome (28; 25.7%), asphyxia (21; 19.3%), prematurity (17; 15.6%), jaundice (15; 13.8%), and meconium aspiration syndrome (12; 11.0%) (Supplementary Table S4). No neonates required oxygen or other respiratory support during the study period. No adverse events due to monitoring were observed and all enrolled neonates remained stable until discharge.

### Total time attached and up-time

**Figure 1:**
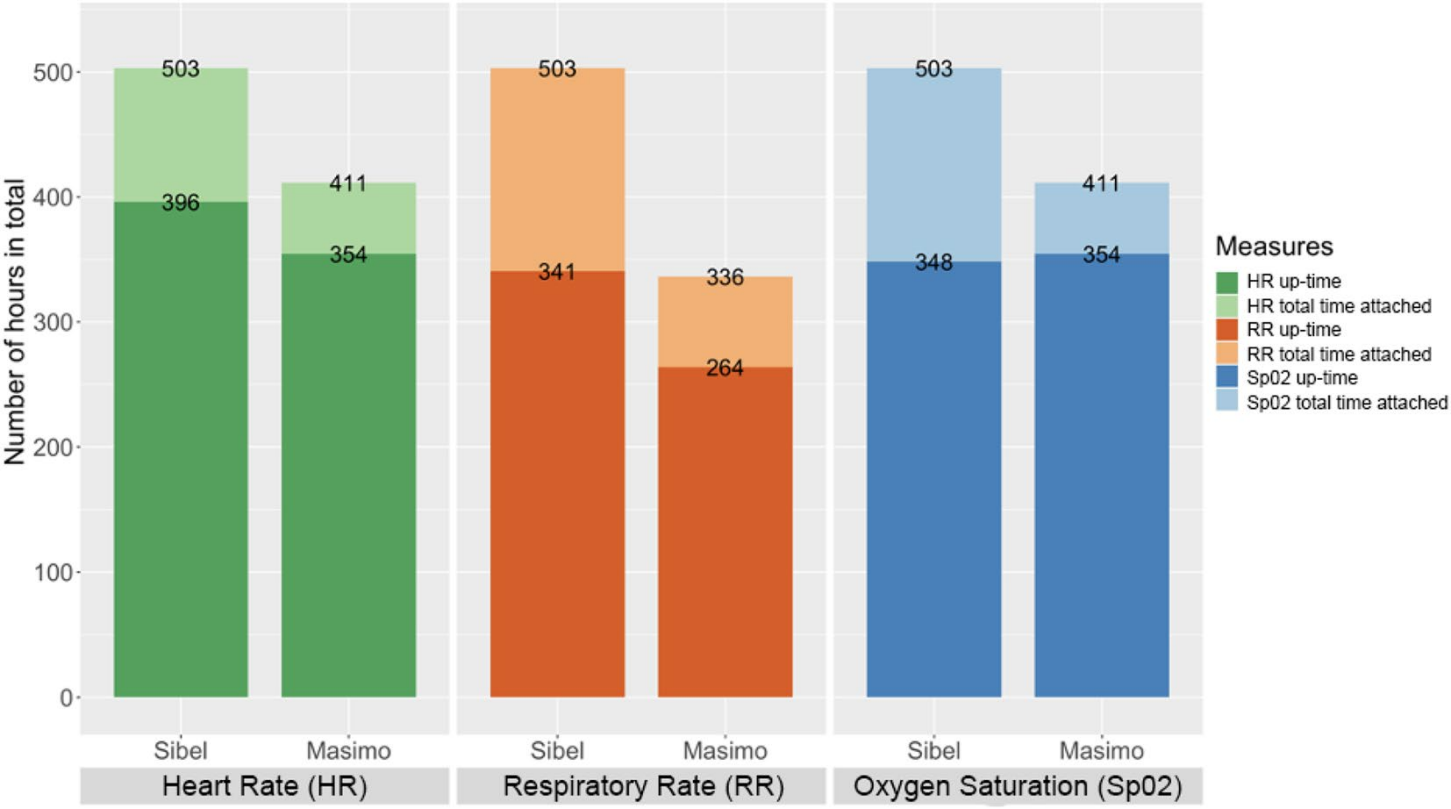
Total time technology attached and up-time.

ANNE exceeded the reference technology’s total and mean up-times per neonate for HR and RR, but not for SpO_2_ (Table 2; Fig. 1). ANNE’s up-time was 42 (11%) h more for HR, 77 (25%) h more for RR, and 6 (2%) h less for SpO_2_ compared to the Rad-97 (Fig. 1). ANNE’s ratio of up-time to total attached time was less than Rad-97’s for HR (0.79 vs 0.86), RR (0.68 vs. 0.79), and SpO_2_ (0.69 vs 0.86).

**Table 2:** Mean up-time per neonate.

	Heart rate		Respiratory rate		Oxygen saturation	
	ANNE	Rad-97	ANNE	Rad-97	ANNE	Rad-97
Mean up-time per neonate in hours [range]	3.6 [0, 7.3]	3.3 [1.2, 5.7]	3.1 [0, 6.1]	2.4 [0, 4.3]	3.2 [0, 6.1]	3.3 [1.2, 5.7]

### Event detection

**Figure 2:**
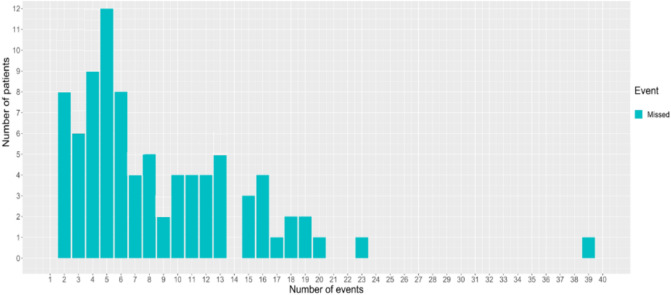
Histogram of oxygen saturation missed events. Missed events were concentrated in select participants.

ANNE’s high HR event detection indicated adequate performance parameters based on sensitivity (100%), specificity (86%), and NPV (100%), but not PPV (31%) (Table 3). ANNE’s high RR event detection also showed adequate performance as demonstrated by the sensitivity (93%), specificity (86%), NPV (85%), and PPV (94%). Similarly, ANNE’s low HR and RR event detection signalled adequate performance for the above parameters with the exception of low PPVs (HR 50%; RR 18%). However, ANNE’s low SpO_2_ event detection demonstrated relatively poor performance among most of the above parameters with the exception of specificity (89%) and PPV (91%). Of note, low SpO_2_ missed events were not normally distributed between cases, but rather concentrated among a small number of neonates (Fig. 2). ANNE’s high temperature event detection performed well across all performance parameters (Table 3).

**Table 3:** Clinical event detection.

	High heart rate		Low heart rate		High respiratory rate		Low respiratory rate		Low oxygen saturation		High temperature	
	Pre	Post	Pre	Post	Pre	Post	Pre	Post	Pre	Post	Pre	Post
**Event**												
True positive	109	109	4	4	1090	1111	3	3	535	1165	1	–
True negative	1426	1633	1661	1661	463	536	1126	1126	464	514	602	–
False positive	240	33	4	4	76	3	14	14	56	6	6	–
False negative	0	0	0	0	81	60	0	0	743	113	3	–
**Statistical summary**												
Accuracy (%)	87	98	100	100	91	96	99	99	56	93	99	–
Sensitivity (%)	100	100	100	100	93	95	100	100	42	91	25	–
Specificity (%)	86	98	99	99	86	99	99	99	89	99	99	–
PPV (%)	31	77	50	50	94	100	18	18	91	100	14	–
NPV (%)	100	100	100	100	85	90	100	100	38	82	100	–
FN:FP	0	0	0	0	1:0.9	1:0.1	0	0	1:0.1	1:0.1	1:2	–

Pre-adjudication (Pre); post-adjudication (Post); positive predictive value (PPV); negative predictive value (NPV); false negative event to false positive event ratio (FN:FP).

Accuracy = (True positive + True negative)/(True positive + True negative + False negative + False positive); Sensitivity = True positive/(True positive + False negative); Specificity = True negative/(True negative + False positive); PPV = True positive/(True positive + False positive); NPV = True negative/(True negative + False negative).

### Event adjudication

Pre- and post-adjudication, there were minimal changes in HR and RR performance parameters with the exception of PPV which improved by 46% post-adjudication for high HR (Table 3). For SpO_2_, a substantial improvement ranging from 9% (PPV) to 49% (sensitivity) was observed across all performance parameters between pre- and post-adjudication of events. Of the 1140 false negative and false positive events, 3 high HR events, 24 high RR events, and 87 low SpO_2_ events were interpreted as clinically significant during adjudication.

### Agreement

**Figure 3:**
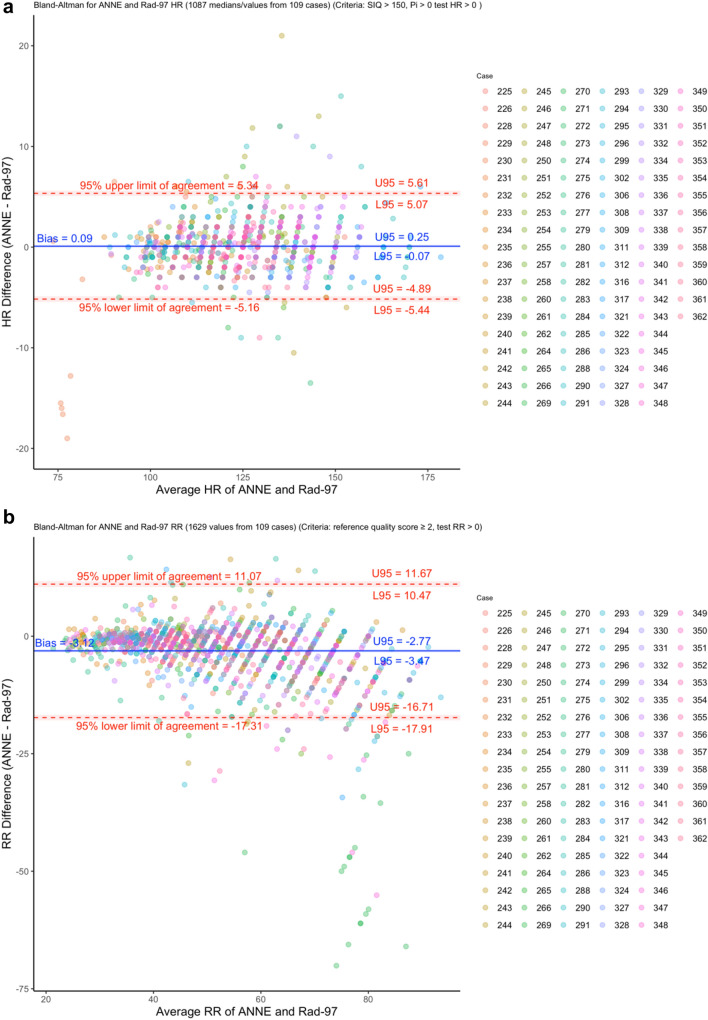

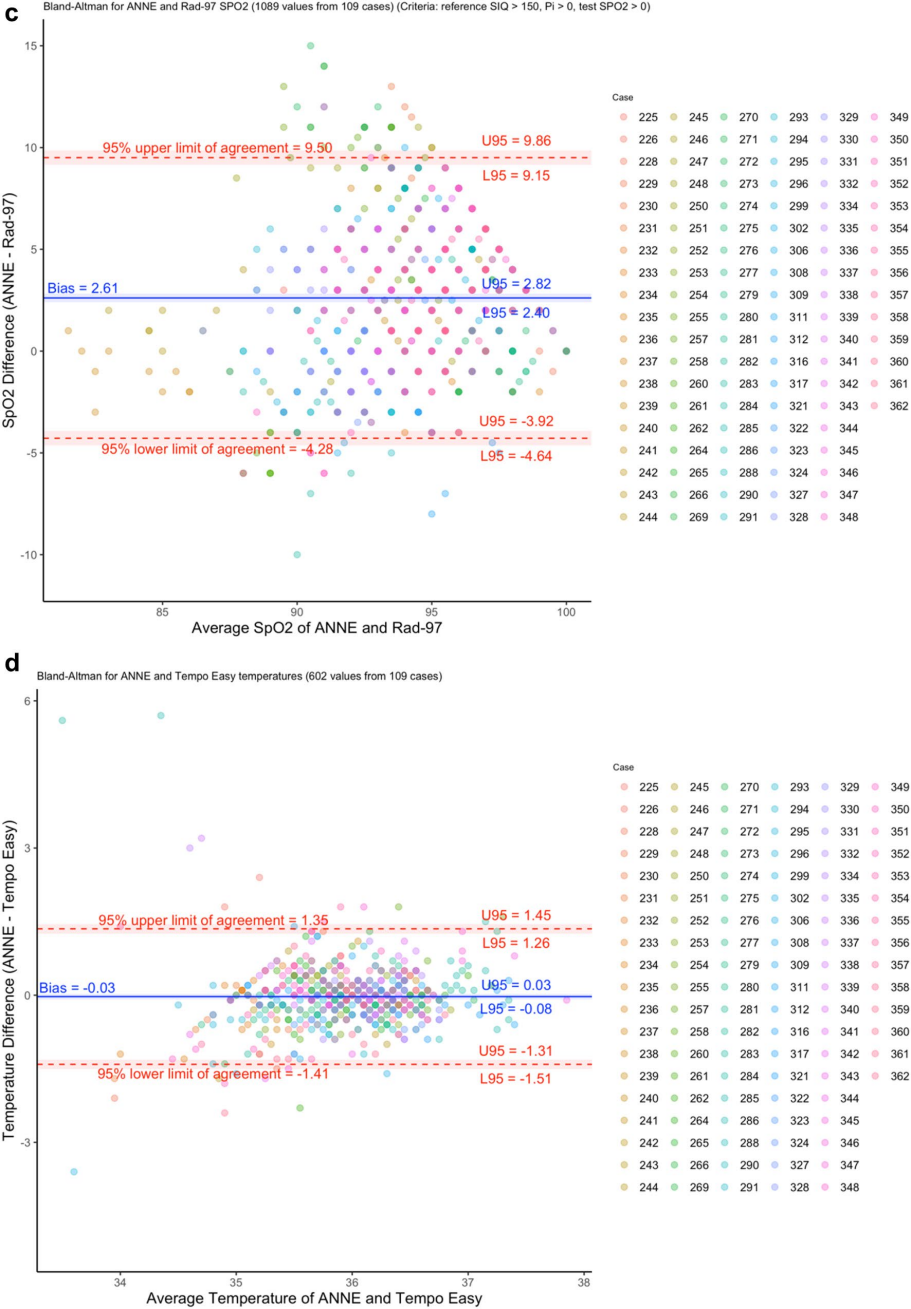
Bland–Altman plots of measured (**a)** heart rate (HR), (**b)** respiratory rate (RR)**, (c)** oxygen saturation (SpO_2_), and (**d)** temperature as measured by ANNE and the reference technologies. Colors indicate which participant neonate is associated with the measurement pair.

HR agreement showed a normalized spread of LOA of 8.4% (Fig. 3a) and RR agreement showed a normalized spread of LOA of 52.2% (Fig. 3b). The a priori-identified limit of 30% for the normalized spread of LOA was met for HR but not RR. SpO_2_ agreement showed a normalized RMSD of 4.4% (Fig. 3c); the adequate a priori-identified limit of 4.5% for the RMSD was met for SpO_2_, but the optimal limit of 3.5% was not met. A sensitivity analysis removing cases where there were at least 10 missed SpO_2_ events revealed a minimal reduction in the RMSD from 4.4 to 4.2% (Supplementary Figure S5). Temperature agreement showed a spread of LOA of 2.8 °C (Fig. 3d) and therefore, met the a priori-identified limit of 4.5 °C.

## Discussion

In an evaluation in a large public maternity hospital in Nairobi, Kenya, we found Sibel’s ANNE to be clinically feasible for some measurement parameters (HR and temperature) but not others (RR and SpO_2_) as demonstrated by the technology’s up-time, clinical event detection performance, and the agreement of measurements compared to those from the reference technologies. Compared to Rad-97’s wired and more invasive technology, wireless ANNE’s up-time was longer because ANNE could continue to monitor the neonate during feeding and kangaroo mother care. However, while HR and temperature event detection were optimal and RR event detection was acceptable, SpO_2_ event detection was not acceptable. Adjudication established that ANNE incorrectly detected RR and SpO_2_ artifacts as clinically significant events during periods of noise, yielding many false positive high RR and low SpO_2_ events. Agreement of neonatal measurements between ANNE and the verified reference technologies showed that HR and temperature measurements met the a priori-identified optimal limits, RR measurements did not, and SpO_2_ measurements met the adequate but not the optimal a priori-identified limits in both the original and sensitivity analyses.

Compared to the findings from a more controlled clinical trial at a better-resourced hospital with a neonatal intensive care unit, there was significant degradation in RR and SpO_2_ agreement in this real-world clinical environment, limiting the clinical feasibility of ANNE to assess these measurements in routine practice^7^. RR agreement degradation may have been due to imprecise sensor placement and/or inadequate skin adherence. The low SpO_2_ missed events were concentrated among a small number of neonates, most likely due to motion artifacts, low perfusion states, and/or inadequate application of the ANNE sensors in individual neonates.

Reliable SpO_2_ measurement and monitoring can be challenging in smaller neonates and those with darker skin pigmentation. Motion artifacts among neonates induce many false alarms, which in turn can cause additional workload for healthcare providers and stress for the neonates and their caregivers as well as potentially impair response times in real critical situations^16–18^. Low perfusion states and variations in breathing can be common in neonates and can degrade pulse oximeter performance^19–21^. Smaller neonatal finger size and a correspondingly smaller pulsatile signal detected by the pulse oximeter sensor may also compromise performance^22–24^. As melanin is a secondary absorber of near-infrared light, darker skin pigmentation may impact pulse oximeter performance and accuracy^25,26^.

In two large cohorts of adults, Black patients had almost three times the frequency of occult hypoxemia undetected by pulse oximetry compared to White patients^27^. However, in a small infant study, no evidence of systematic bias in pulse oximetry measurement based on skin pigmentation was found in 36 patients with hypoxemia^28^. In another study of 294 neonates less than 32 weeks gestation, SpO_2_ overestimation measured by mean bias was 2.4-fold greater among Black preterm neonates and resulted in greater occult hypoxemia^26^. Validation studies of new medical technologies need to include diverse populations^29^.

Previous studies have evaluated the accuracy of single parameter monitoring technologies under controlled experimental conditions or have performed qualitative feasibility assessments^30,31^. In our study, we utilized a comprehensive and integrated approach that included real-world accuracy evaluations combined with performance metrics such as up-time and event detection that are often important in critical clinical situations. Solely focusing on the accuracy may not appropriately reflect the poor performance in event detection in individual cases. Regulatory approval typically requires controlled experiments looking at agreement or accuracy but does not consider real-world applications or event detection. Performing well in a controlled accuracy evaluation, while important, does not necessarily ensure a medical technology will perform well in clinical practice and have real-world impact.

Limitations to our evaluation, analyses, and adjudication included the relatively arbitrary a priori signal quality limits we selected to mitigate confusion that could have arisen if poor quality signals were used for event detection. To ensure comparable event detection, we evaluated the technologies in real-time even though we analyzed the data later. Limiting the generalizability of our evaluation was a relatively healthy study population with few critically ill neonates included and infrequent life-threatening events recorded. While comparing ANNE’s electrocardiogram-derived HR to Rad-97’s photoplethysmogram-derived HR may contribute to increased uncertainty in these comparisons, HR estimation has been shown to be well preserved from the photoplethysmogram^32^. We limited our comparison to the detection of low RR and did not evaluate the detection of apnea since ANNE did not have an apnea algorithm. Overall, clinically significant events were relatively uncommon despite the sustained duration of monitoring, and we did not permit ANNE or the reference technologies to generate alarms that may have impacted clinical outcomes. ANNE would benefit from being further evaluated in different populations (e.g., preterm and more critically ill neonates) and settings with assessment of clinical outcomes and/or impact. To be clinically feasible, safe, and effective in improving quality of neonatal care, a monitoring technology needs to avoid excessive alarms (and alarm fatigue) resulting from false positive events and to suppress transient artifacts without missing clinically significant true positive events. Typically, this could be accomplished by employing certain false alarm limiting strategies such as an appropriate delay before alarm generation. Exploring threshold and adaptive alerts provided by the technology, assessing barriers and facilitators to adoption and use, and conducting costing studies will be key to ANNE’s development and if successful, to its uptake and scale-up.

There were additional technology constraints. ANNE experienced intermittent difficulty turning on after more than one week without use and a software bug resulted in some corruption of data and data loss. The wireless Bluetooth technology required daily charging of batteries, with regular checks to mitigate against data loss. Furthermore, the battery life degraded over the study period. Another potential limitation of wireless technology may include electrical interference from other devices in the vicinity; however, this was not experienced in this study. For temperature measurement, both ANNE and Tempo Easy’s technology measured skin surface temperature, which is dependent on the environmental room temperature, and thus, may not be accurate in detecting hypothermia. The measurement of core temperature is critical in neonates due to their large surface area which accelerates heat loss. Hypothermia is a common presenting sign for other severe physiological disturbances such as sepsis. For this reason, we did not set an event threshold for low temperature, but we acknowledge that monitoring core body temperature in neonates may be critically important. The Rad-97 also had constraints, including that it was relatively heavy with cumbersome sensory cables and cords, and prone to overheating when the air vent was covered. Furthermore, its sensors were not reusable and the nasal cannula was more frequently removed, limiting the technology’s up-time.

Despite the limitations and technology constraints, the study results from this and a previous accuracy evaluation in Kenya indicate that ANNE appears to be clinically feasible for wirelessly monitoring HR and temperature but not RR and SpO_2_ when compared to the more invasive Rad-97 reference technology^7^. Studies of monitoring technologies in neonates have largely been limited to high-income countries and would benefit from diversification of study populations. Established methods for continuous monitoring exist, but factors such as invasiveness, time-consuming application, and high cost have contributed to feasibility concerns in resource-constrained settings. Novel neonatal multiparameter continuous monitoring technologies such as ANNE may be able to address these concerns; however, more work needs to be done regarding ANNE’s RR and SpO_2_ measurements.

## Supplementary Information

Supplementary Information.

## Data Availability

Access to data will be provided to researchers subject to submission of a research proposal and signing a Data Use Agreement. Interested researchers can request access to the data at https://doi.org/10.25934/PR00007550 by creating a Vivli account and submitting a request. Data will be available from September 2022.
