# Supplementary material for: Clinical feasibility of an advanced neonatal epidermal multiparameter continuous monitoring technology in a large public maternity hospital in Nairobi, Kenya
Source: Sci Rep. 2022 Jul 9;12:11722. doi: 10.1038/s41598-022-16051-3 (PMC9271033; doi:10.1038/s41598-022-16051-3)
Supplement: Supplementary file 1 — Supplementary Information. [file 41598_2022_16051_MOESM1_ESM.docx]

**Supplementary material**

| **Supplementary Figure S1.** A computer rendering of the Sibel Advanced Neonatal Epidermal (ANNE) system investigational vital signs monitoring platform. The system consists of a chest sensor (L) and a limb sensor (R). The system can measure heart rate, respiratory rate, oxygen saturation and skin surface temperature. |
| --- |
| 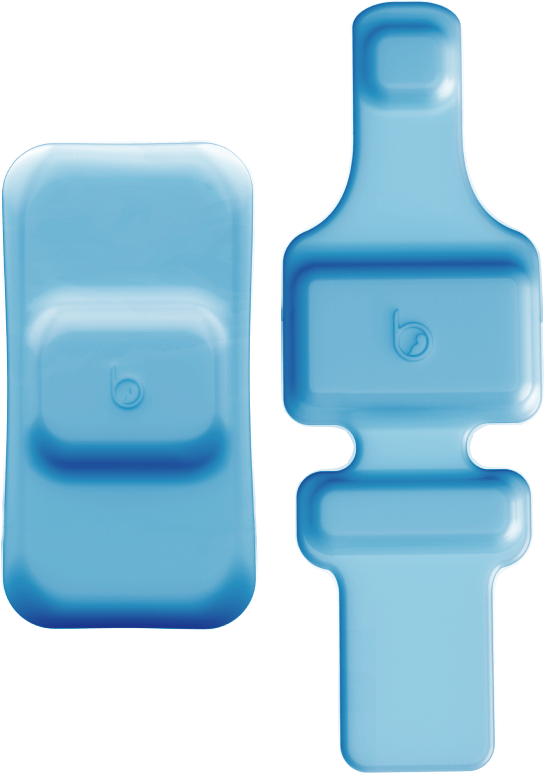 |

**a
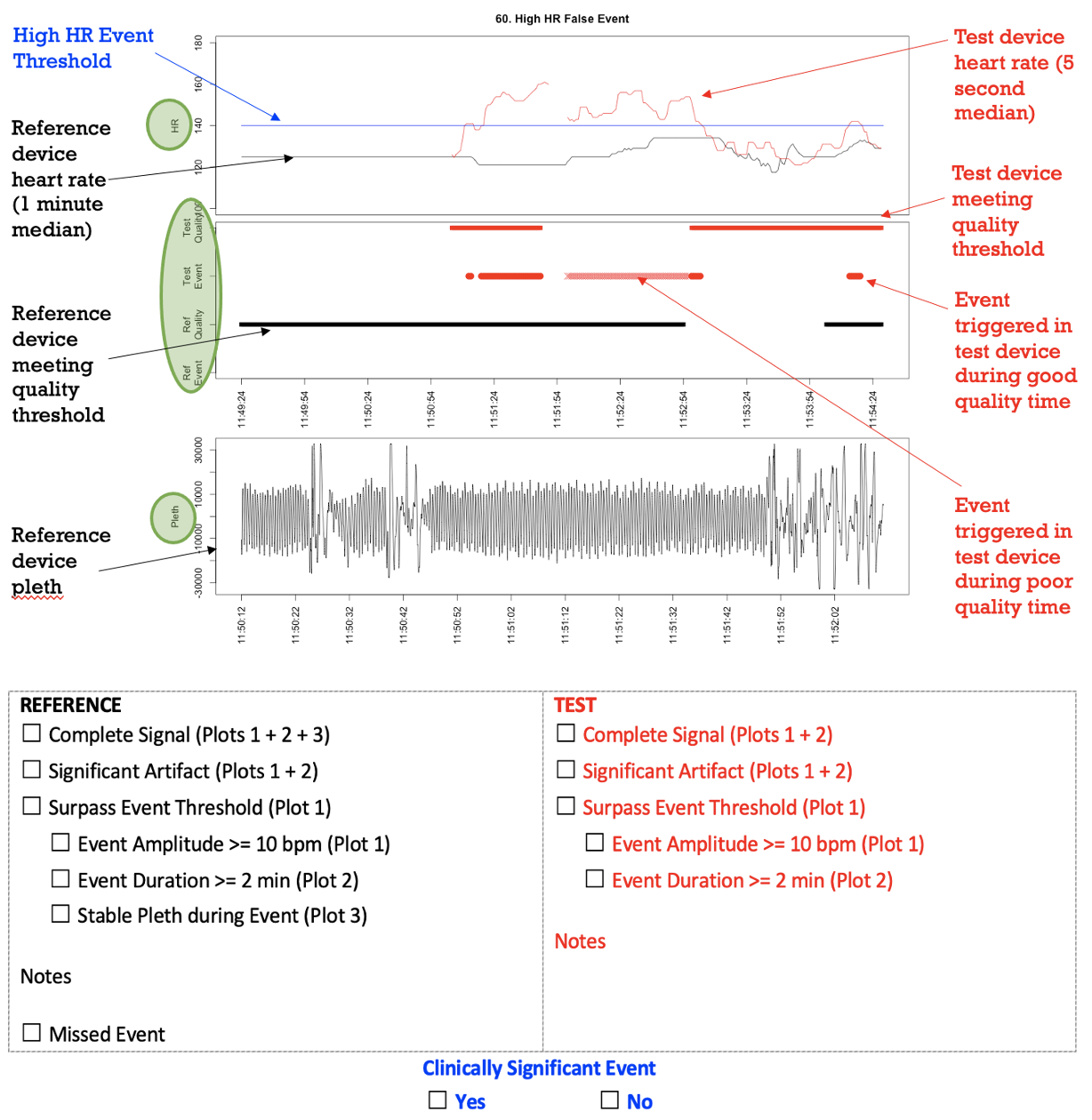
**

**b**

**
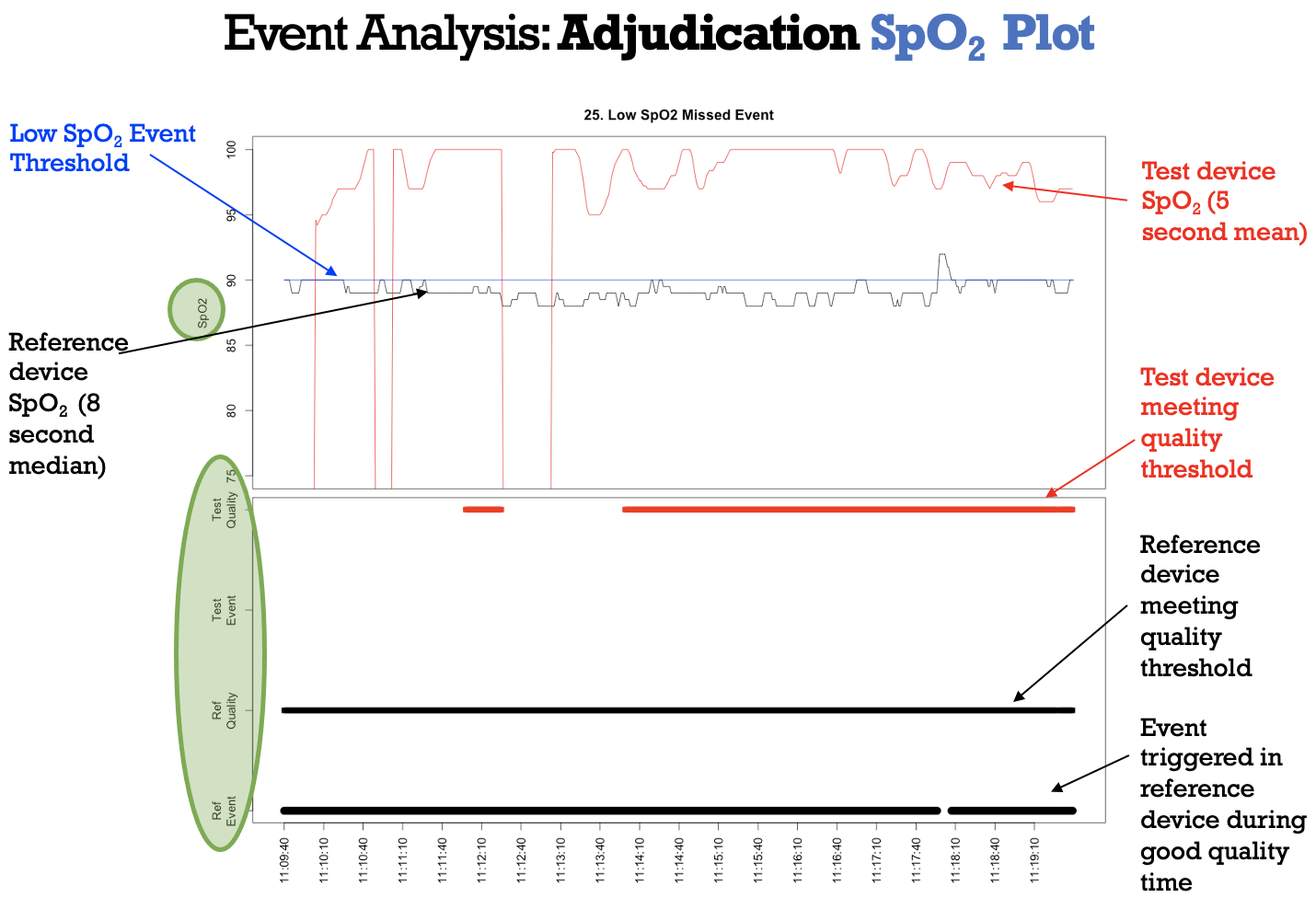
**

**
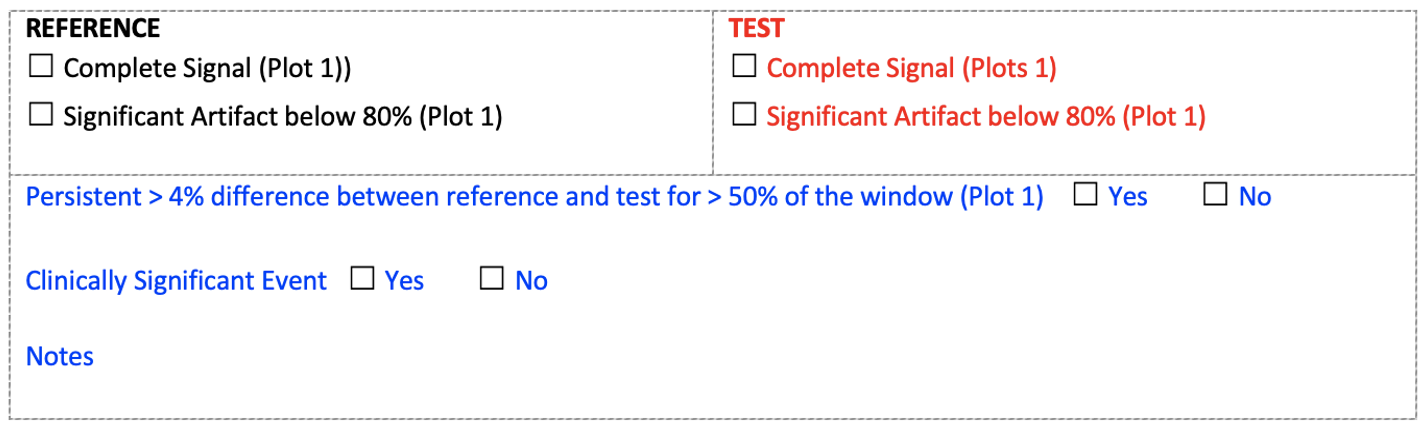
**

**Supplementary Figure S2.** **a)** Sample adjudication form for heart rate. Respiratory rate form resembles heart rate except capnography replaces the plethysmograph. **b)** Sample adjudication

form for oxygen saturation.

Neonates enrolled (n=114)

Neonates’ data excluded (n=5)

♦  Insufficient data recording length (n= 5)

Neonates’ data included in analysis (n=109)

**Supplementary Figure S3.** Flow diagram showing enrolled neonates.

| **Primary diagnosis** | **Number** | **Percent** |
| --- | --- | --- |
| Sepsis/suspected sepsis | 26 | 23.9 |
| Respiratory distress syndrome | 20 | 18.3 |
| Asphyxia | 19 | 17.4 |
| Prematurity | 12 | 11.0 |
| Meconium aspiration syndrome | 7 | 6.4 |
| Hypoxia | 6 | 5.5 |
| Jaundice | 6 | 5.5 |
| Macrosomia | 3 | 2.8 |
| Dehydration | 2 | 1.8 |
| Low birthweight | 2 | 1.8 |
| Mother VDRL positive | 2 | 1.8 |
| None | 2 | 1.8 |
| Hepatitis B exposed | 1 | 0.9 |
| Poor latching | 1 | 0.9 |
| Total | 109 | 100 |
| **Secondary diagnosis** |  |  |
| Jaundice | 9 | 22.0 |
| Respiratory distress syndrome | 8 | 19.5 |
| Meconium aspiration syndrome | 5 | 12.2 |
| Prematurity | 5 | 12.2 |
| Sepsis/suspected sepsis | 3 | 7.3 |
| Asphyxia | 2 | 4.9 |
| Hypothermia | 2 | 4.9 |
| Convulsions | 1 | 2.4 |
| Dehydration | 1 | 2.4 |
| Hypoglycemia | 1 | 2.4 |
| Low birthweight | 1 | 2.4 |
| Macrosomia | 1 | 2.4 |
| Ophthalmia neonatorum | 1 | 2.4 |
| Periorbital sepsis | 1 | 2.4 |
| Total | 41 | 100 |

**Supplementary Table S4.** Primary and secondary diagnoses during enrolled neonates’ current hospitalizations (per hospital medical chart).

**
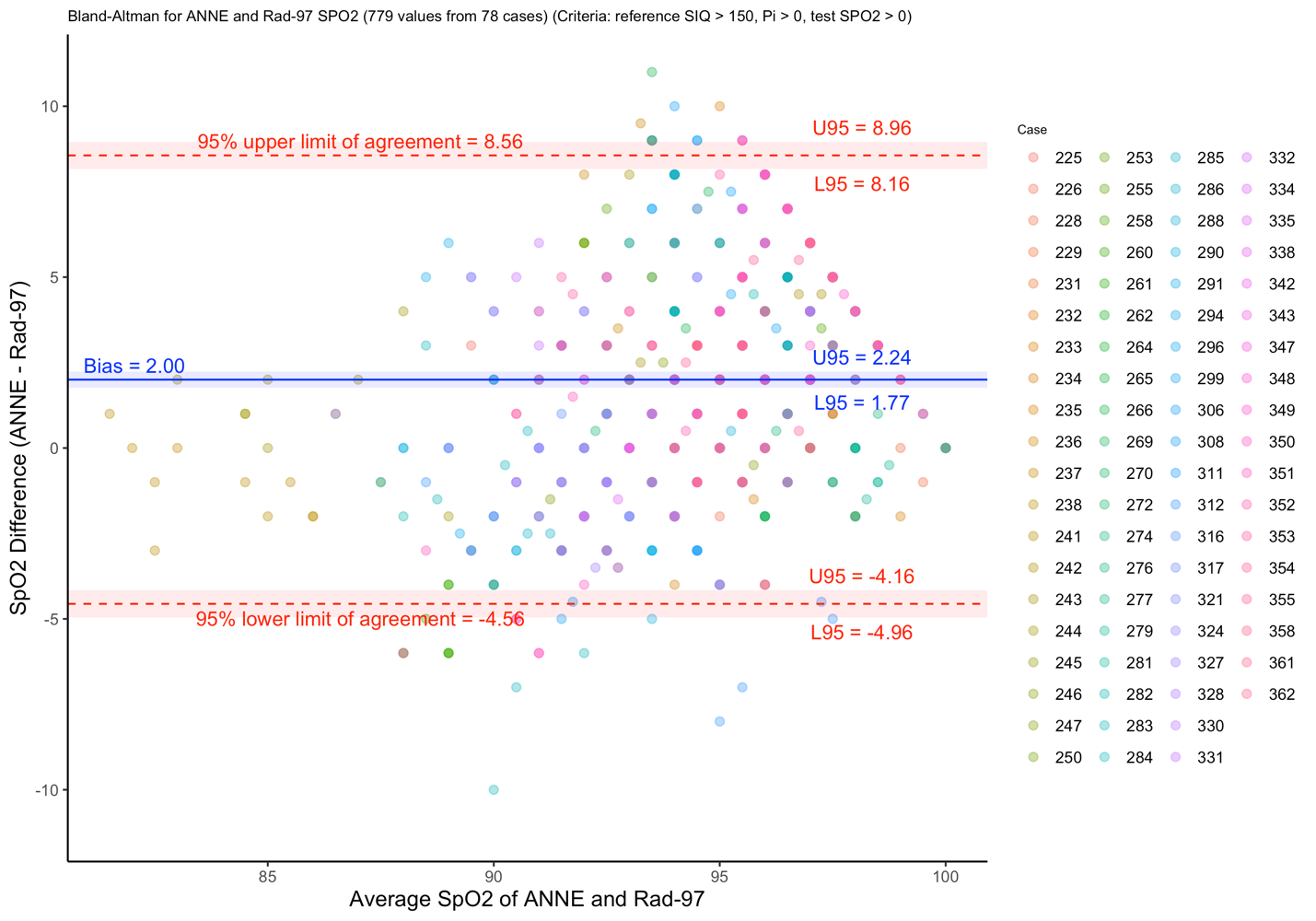
**

**Supplementary Figure S5.** Bland-Altman plots of oxygen saturation (SpO_2_) as measured by ANNE and Rad-97 technologies. Thirty-two cases where there were at least 10 missed SpO_2_ events were removed. Colors indicate which participant neonate is associated with the measurement pair.
